# The entomological impact of passive metofluthrin emanators against indoor *Aedes aegypti*: A randomized field trial

**DOI:** 10.1371/journal.pntd.0009036

**Published:** 2021-01-26

**Authors:** Gregor J. Devine, Gonzalo M. Vazquez-Prokopec, Wilbert Bibiano-Marín, Norma Pavia-Ruz, Azael Che-Mendoza, Anuar Medina-Barreiro, Josue Villegas, Gabriela Gonzalez-Olvera, Mike W. Dunbar, Oselyne Ong, Scott A. Ritchie, Thomas S. Churcher, Oscar D. Kirstein, Pablo Manrique-Saide

**Affiliations:** 1 Mosquito Control Laboratory, QIMR Berghofer Medical Research Institute, Brisbane, Queensland, Australia; 2 Department of Environmental Sciences, Emory University, Atlanta, Georgia, United States of America; 3 Unidad Colaborativa de Bioensayos Entomológicos, Campus de Ciencias. Biológicas y Agropecuarias, Universidad Autónoma de Yucatán, Mérida, Yucatán, México; 4 Centro de Investigaciones Regionales Hideyo Noguchi, Universidad Autónoma de Yucatán, Mérida, México; 5 College of Public Health, Medical and Veterinary Sciences, James Cook University, Queensland, Cairns, Australia; 6 Department of Infectious Disease Epidemiology, MRC Centre for Global Infectious Disease Analysis, Imperial College London, London, United Kingdom; University of Wisconsin Madison, UNITED STATES

## Abstract

**Background:**

In the absence of vaccines or drugs, insecticides are the mainstay of *Aedes*-borne disease control. Their utility is challenged by the slow deployment of resources, poor community compliance and inadequate household coverage. Novel application methods are required.

**Methodology and principal findings:**

A 10% w/w metofluthrin “emanator” that passively disseminates insecticide from an impregnated net was evaluated in a randomized trial of 200 houses in Mexico. The devices were introduced at a rate of 1 per room and replaced at 3-week intervals. During each of 7 consecutive deployment cycles, indoor resting mosquitoes were sampled using aspirator collections. Assessments of mosquito landing behaviours were made in a subset of houses. Pre-treatment, there were no differences in *Aedes aegypti* indices between houses recruited to the control and treatment arms. Immediately after metofluthrin deployment, the entomological indices between the trial arms diverged. Averaged across the trial, there were significant reductions in Abundance Rate Ratios for total *Ae*. *aegypti*, female abundance and females that contained blood meals (2.5, 2.4 and 2.3-times fewer mosquitoes respectively; P<0.001). Average efficacy was 60.2% for total adults, 58.3% for females, and 57.2% for blood-fed females. The emanators also reduced mosquito landings by 90% from 12.5 to 1.2 per 10-minute sampling period (P<0.05). Homozygous forms of the pyrethroid resistant kdr alleles V410L, V1016L and F1534C were common in the target mosquito population; found in 39%, 24% and 95% of mosquitoes collected during the trial.

**Conclusions/Significance:**

This is the first randomized control trial to evaluate the entomological impact of any volatile pyrethroid on urban *Ae*. *aegypti*. It demonstrates that volatile pyrethroids can have a sustained impact on *Ae*. *aegypti* population densities and human-vector contact indoors. These effects occur despite the presence of pyrethroid-resistant alleles in the target population. Formulations like these may have considerable utility for public health vector control responses.

## Author summary

Insecticidal control tools are heavily relied on for the control of mosquito-borne viruses such as dengue, chikungunya and Zika, but the logistics associated with conventional insecticide use (e.g. space sprays and residual formulations) are challenging. Considerable time and resources are required to treat household interiors; an impediment exacerbated by the difficulty in gaining entrance to households, and sometimes by limited compliance in the community. Another constraint to effective insecticide use is that many mosquito populations are resistant to the chemicals used. Volatile pyrethroids, exhibiting both lethal and behavioural effects on mosquitoes are available in formulations that release insecticides passively to the air, at room temperature. These may be suitable for deployment in houses with the aim of creating “bite-free” spaces. By removing the need for conventional application methods, these devices might be rapidly deployed with minimum disruption to households. This is the first large-scale, randomized control trial to evaluate the entomological impacts of volatile pyrethroids in an urban environment. Using metofluthrin as an example, we confirm that some formulations have a significant impact on *Aedes aegypti* densities and landing behaviour indoors. These effects occur despite the presence of pyrethroid-resistance alleles associated with conventional insecticide resistance.

## Introduction

In the absence of vaccines or drugs for combating urban, *Aedes*-borne viruses (ABV) such as dengue, Zika and chikungunya, insecticides remain the mainstay of disease and vector control programs. *Aedes aegypti*, the primary urban vector of ABVs, has a predominantly endophilic and endophagic behaviour [1,2] and outbreaks of ABVs are therefore most effectively tackled with rapid, insecticide-based campaigns that focus on household interiors. These aim to kill viremic mosquitoes and reduce adult female survival. Generally, vector control interventions are implemented in response to reports of symptomatic ABV infections and involve the application of insecticides outdoors (e.g., vehicle-mounted ULV spraying) and indoors (e.g., indoor space spraying, targeted residual treatments) across large numbers of households. Although significant entomological impacts may result [3–5] this approach relies on considerable human resources, logistical support and community compliance to achieve effective coverage. A major barrier to effective implementation during outbreaks is that the rapid and extensive coverage of households is challenged by the time it takes spray teams to treat interiors, the difficulty of gaining entrance, and community compliance [6,7]. Another major obstacle is that many mosquito populations are resistant to the insecticides used for control; particularly the pyrethroids [5,8].

Volatile insecticides, mainly synthetic pyrethroids, are widely available as constituents of consumer products that claim to reduce adult mosquito nuisance. Some volatile pyrethroid formulations are available as components of powered devices that use heat or a fan to assist the release of chemical [9–11], while others rely on the passive release of insecticide, facilitated at room temperature, by natural air flows [12]. Various iterations of these devices are marketed for indoor and outdoor use and emphasize impacts on biting, rather than on knock down and mortality [13]. Similar devices, that release volatile insecticides from a point source (here termed emanators) could offer a potential solution to the issues of speed and compliance that challenge the application of conventional insecticide formulations indoors. The contained, portable nature of these emanators may make them suitable and safe for deployment by the community or by vector response personnel with little training.

The use of volatile insecticides to reduce vector-borne disease transmission is currently captured by the World Health Organization’s Vector Control Advisory Group under the “spatial repellent” product class. These products interrupt human–vector contact through the behavioural impacts of airborne chemicals [14,15]. This definition encompasses true repellency (movement away from a chemical stimulus), interference with host detection, and any other non-lethal impact on biting and feeding. Spatial repellency is considered distinct from contact irritancy or direct toxicity [16] but it is evident that volatile insecticides will exert a range of impacts against insects depending on air concentration and proximity to the chemical source. At low concentrations some effects will be behavioural, while at higher doses the impacts may include sub-lethal poisoning and direct toxicity. Passive emanators incorporating the polyfluorinated, volatile pyrethroids transfluthrin and metofluthrin are gaining attention as public health tools against mosquito-borne diseases mainly for malaria control [16–18] but more recently to manage ABVs [13,19,20]. To date, the entomological impacts of these volatiles have largely been evaluated under laboratory or semi-field conditions [17,19,21,22]. Few field trials have addressed their impacts on indoor-biting mosquitoes [18,23], and none have been conducted on *Ae*. *aegypti*. The extent to which transfluthrin or metofluthrin are effective against *Ae*. *aegypti* under operational conditions therefore remains untested, although other trials, most notably in the Peruvian Amazon, are underway [15]. Moreover, the degree to which the efficacy of volatile insecticides is impacted by the presence of the metabolic or sodium channel mutations that confer resistance to more conventional pyrethroids remains unclear [24–26].

In this study, we report the findings of a randomized field trial evaluating the entomological impact of passive emanators containing the volatile pyrethroid metofluthrin (10% active ingredient by weight) against urban *Ae*. *aegypti* in the state of Yucatán, Mexico. These emanators require no power or heat and are estimated to remain effective over a three-week replacement cycle [13]. Metofluthrin (2,3,5,6-tetrafluoro-4-methoxymethylbenzyl (E,Z) (1R,3R)-2,2- dimethyl-3-(prop-1-enyl) cyclopropane carboxylate) is a volatile pyrethroid with a vapor pressure >200 times that of permethrin. In vapor phase, dependent on dose, metofluthrin affects behaviour and mortality in *Ae*. *aegypti* adults [13]. Our trial used households as the units of treatment and analysis and evaluated the entomological impacts of continuous (cyclical) emanator deployment using four endpoints: indoor *Ae*. *aegypti* adult abundance, female abundance, blood-fed abundance and estimates of *Ae*. *aegypti* landing behaviour.

## Materials and methods

### Ethics statement

This study was approved by the ethics and safety committees of the Ministry of Health, Yucatan, Emory University and QIMR Berghofer. Written informed consent was obtained for each head of recruited households (≥ 18 years old) at the beginning of the study.

### Study area

**Fig 1:**
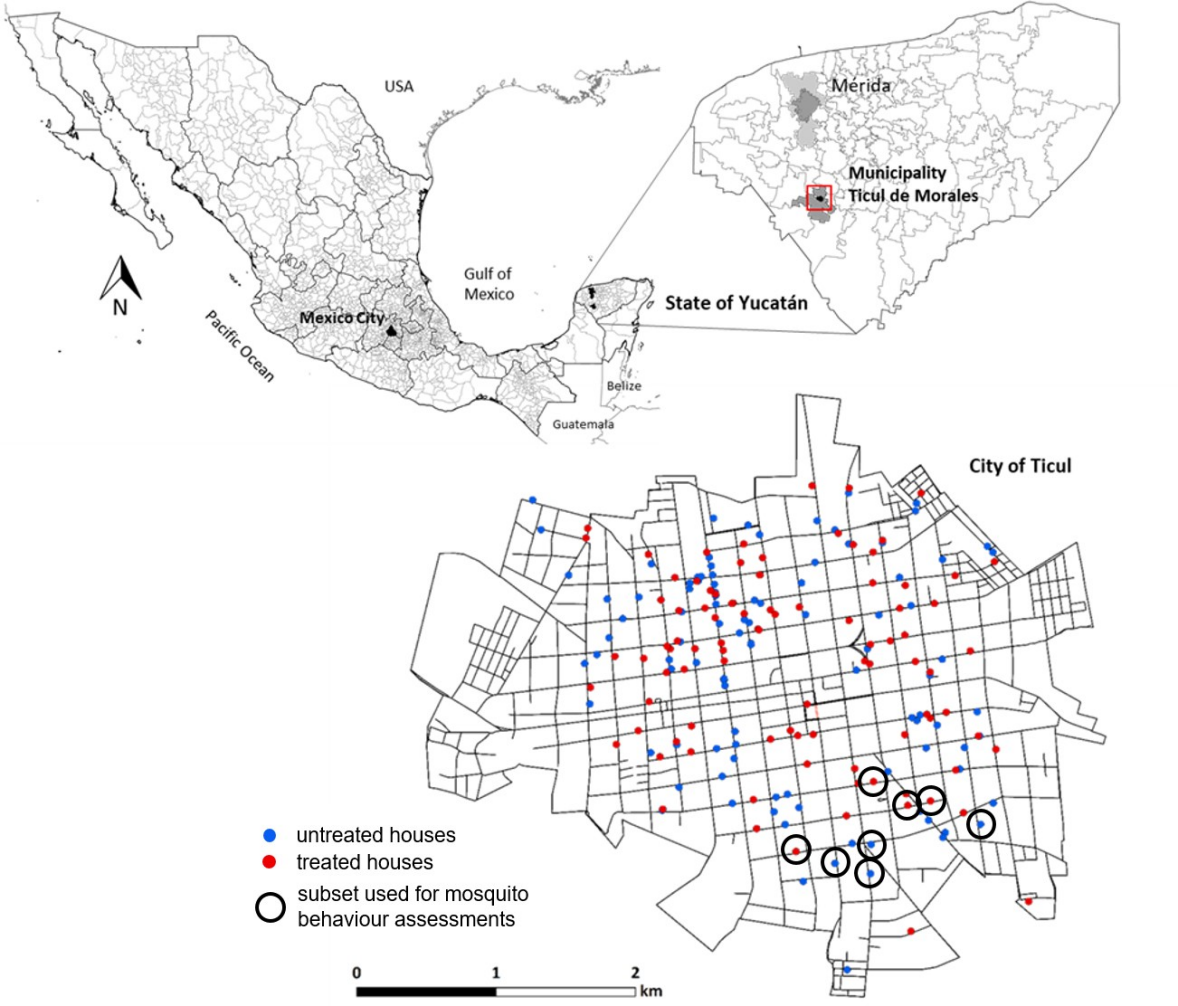
Map of the city of Ticul, Yucatán state (Mexico) showing the random distribution of metofluthrin-treated houses (red dots), untreated controls (blue dots) and the subset of houses selected to assess mosquito landing behaviour (circled dots).

The study was conducted in the city of Ticul, municipality of Ticul de Morales, Yucatan State, Mexico (Fig 1). Ticul (20°24′ N/ 89°32′ W) is 25 m above sea level and, and covers an area of 10.8 km^2^ with a population of 32,769 people (Instituto Nacional de Estadística y Geografia) [27]. The climate is classified as tropical savanna with dry-winter characteristics (Aw using the Koppen classification [28]). It has a summer rainy season from May through November (ca. 1,065 mm average). Dengue and other *Aedes*-borne viruses are endemic throughout Yucatan State, with peak dengue transmission typically occurring between July and November [6,29,30]. Ticul experiences considerable dengue transmission, evidenced by longitudinal cohort studies, with 81% sero-prevalence in ≤ 60 year-olds [31] and increasing sero-prevalence from 30% in ≤ 5 year-olds to 68% in 9–15 year-olds [32].

### Metofluthrin emanators

**Fig 2:**
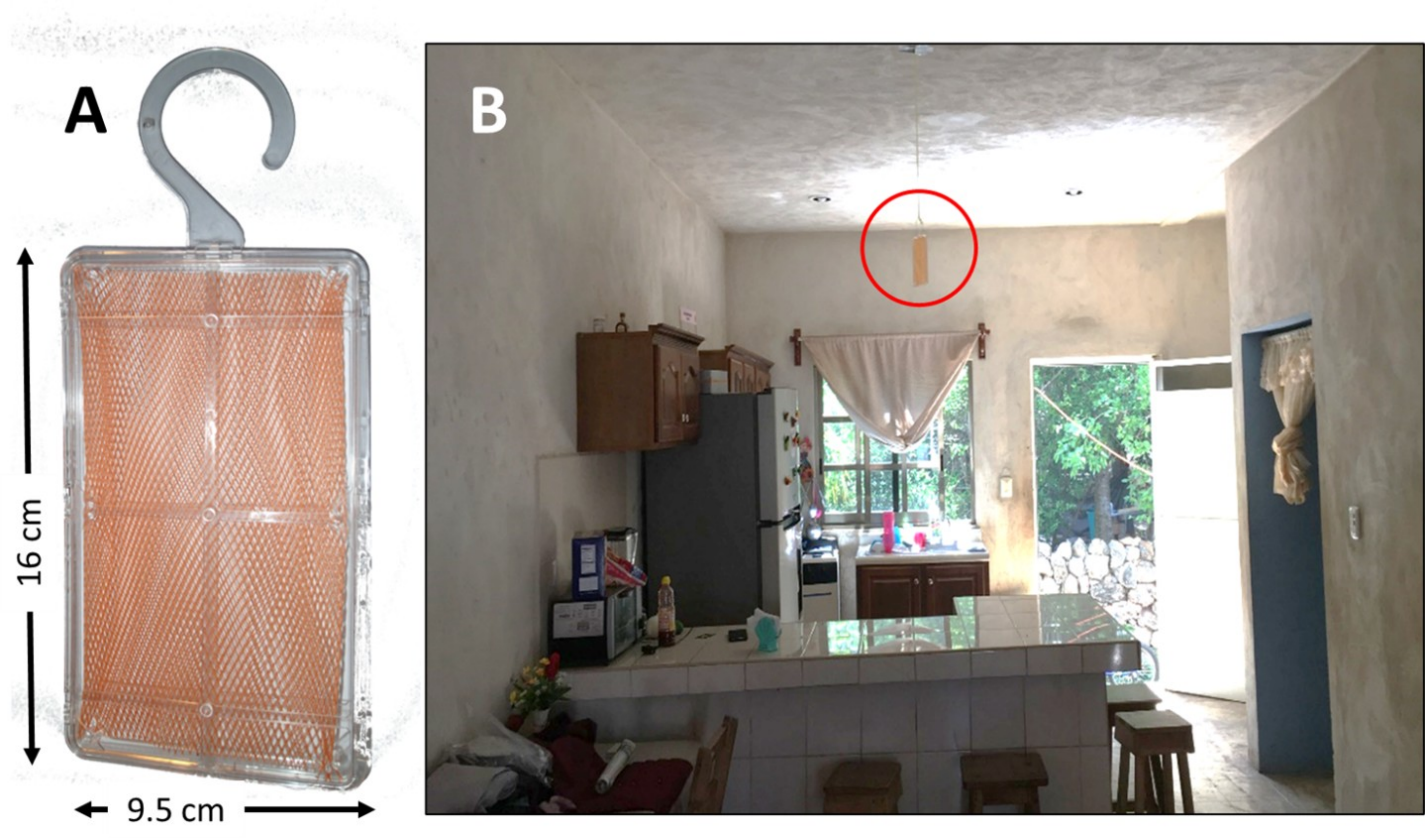
Placement of the metofluthrin passive emanators in a room of treatment house. The emanators consist of metofluthrin impregnated mesh contained in a plastic housing (A). Metofluthrin impregnated emanators were hung from ceilings, above head height, to keep them clear of routine household movement and activity. The emanator in this room is circled in red (B).

The passive emanators consist of a methacrylate polymer net impregnated with 10% w/w (ca. 0.217 g) of the synthetic, volatile pyrethroid metofluthrin (Sumitomo Chemical Company Ltd. Chuo-ku, Tokyo, Japan). Various iterations of this formulation are currently registered in Australia, Singapore, Malaysia and Thailand (e.g. Australian APVMA approval 70086/62469, Singapore NEA approval I-AmbEN/048/0829) where they are sold as domestic consumer products for the prevention of mosquito bites indoors. The impregnated net is contained within a 95 mm x 160 mm plastic holder (Fig 2A) designed to be hung or placed in rooms with gentle air circulation to encourage volatilization (Fig 2B). Strong airflows will dilute the device’s impact [13]. Previous laboratory and semi-field experiments have shown emanators to be highly effective against pyrethroid-susceptible *Ae*. *aegypti* indoors [13,19,33], remaining effective for 3 weeks after deployment [13]. In the context of our study, we refer to these devices as “metofluthrin emanators”.

### Experimental design

To evaluate the entomological impact of the metofluthrin emanators, we implemented a two-arm, unrestricted randomized trial design with individual households as the units of treatment and analysis. From an initial cohort of 200 households, 100 were randomly assigned to treatment with metofluthrin emanators and the remainder were assigned as as untreated controls (Fig 1). Blank placebos were not available for use in control houses but would have been of limited value as experienced operators can distinguish batches of treated and untreated materials though slight changes in odour. Baseline entomological data were used to confirm that we had sufficient power in our trial to determine a 50% decrease in *Aedes* indices. Using negative binomial dispersion parameters in the PASS software package (Power Analysis and Sample Size Software 2019 NCSS, Kaysville, Utah, USA) we demonstrated 85% power for this design.

### Recruitment and inclusion criteria

An experienced social sciences team, familiar with the community in Ticul through the “Familias sin dengue” program [32], randomly selected households from within the town’s perimeter and invited them to take part in the project. Potential participating heads of household were provided with verbal and written instructions about the installation of the product and a demonstration of the process was offered. After discussion, the household-head was asked if they would consent to repeated entomological assessments, the regular replacement of devices, and assignation of their household to one of the two study arms. It was made clear that participants could end their involvement at any point, without penalty. S1 Table shows the basic construction characteristics of enrolled households in the two study arms. Dwellings were typically single-story (98%) concrete (77%) houses with a front and/or backyard (75%) defined by a cement, stone, or wire perimeter. The windows and doors of most houses lacked properly installed mosquito screens, with incomplete installation (38%) or no screens at all (62%).

The study was designed to quantify the efficacy of metofluthrin emanators in reducing indoor *Ae*. *aegypti* abundance during continuous deployment over a typical ABV transmission season. The routine vector control program by the local Ministry of Health, implemented in response to disease outbreaks, is truck-mounted ULV spraying of chlorpyriphos, larviciding with methoprene and indoor space spraying with chlorpyrifos in the premises of symptomatic cases reported to the healthcare system. However, during the period of our trial there was very little ABV recorded in Ticul and no vector control activity by the MOH (see discussion). The trial included a single entomological baseline measure (to quantify potential differences between treatment and control arms) at the start of the study, followed by the installation and cyclical replacement of emanators at three-week intervals. Entomological surveys began in treatment and control arms within 2–5 days of metofluthrin emanator installation. Baseline measures began on 30^th^ April 2018. Emanator deployment cycles began on 28^th^ May 2018 and ended 31^st^ October 2018 (see S2 Table for the dates of each deployment cycle).

An experienced field team from the Universidad Autónoma de Yucatán—Unidad Colaborativa de Bioensayos Entomológicos (UADY-UCBE) oversaw emanator installation and interacted with householders to identify optimal installation locations (Fig 2). Basic measures of the indoor living space were obtained for each house, including total area, building materials, area of each room and the number of doors and windows. These measures were used to guide the optimum installation. Guided by prior publications on potential deployment rates, the team aimed to install one emanator per room or, for larger spaces, one emanator per 3–4 meter square (9–16 m^2^) [13,19]. Emanators were not installed in hallways or corridors. Emanators were hung from ceilings, above head height, to keep them clear of routine household activity. Devices were attached using existing fixtures (nails, hooks, light fittings) or wire hooks with an adhesive base (Command, Mexico). On average, 5.1 ± 0.08 (mean ± SEM) emanators were installed per house, with the first deployments in May 2018. Without exception the team was allowed access to all rooms in every recruited household.

### Entomological sampling

All installations and assessments were conducted during standard working hours: 8 am–noon and 2 pm—6 pm. Adult indoor resting mosquitoes were collected from all rooms within every house recruited to the trial using Prokopack aspirators [34]. Two field collectors aspirated mosquitoes from each house for a total of 30 minutes, distributing that time evenly across all rooms. We have shown that 30 min collections capture over 95% of all resting adults found indoors [35]. Depending on resources and ease of access it took about two weeks to complete these entomological evaluations across the 200 houses recruited to the trial. This meant that all evaluations could be conducted within the three-week replacement cycle for the emanators. Mosquito samples were processed on the day of collection. Date, house identification number, species, sex and presence or absence of a full or partial blood meal were recorded. Following characterization, individual *Ae*. *aegypti* were preserved in vials with 1 mL RNAlater (Thermo Fisher Scientific, Waltham, MA, USA) and 0.1% Tween 20 (Sigma-Aldrich Co.). Those samples were refrigerated until they could be screened for the presence or absence of point mutations with a known role in conventional pyrethroid resistance (see below).

### Mosquito landing behaviour

The effect of metofluthrin emanators on host-seeking *Ae*. *aegypti* was assessed in two sub-evaluations that each used 8 houses (4 untreated and 4 treated) with high baseline mosquito numbers. These houses were selected on the basis of having high baseline entomological indices. Evaluations of treatment impact were conducted during the first round of installation, immediately following emanator placement. Briefly, experienced field workers quantified landings by sitting in one room of a selected household with one leg exposed. They were otherwise fully protected. As mosquitoes landed on their exposed skin, the operator waved them away with their hands. This method of assessing mosquito activity prevents biting [36] and does not confound results by the sequential removal of mosquitoes during the testing period. Measurements were performed by teams of three, with each member conducting counts in a different living space or bedroom. Each assessment lasted for 10 minutes. Measures were made 10 min, 30 min, 24 hr, 48 hr and 72 hr after installation of the emanators. Observers were randomized between rooms and time points. Data was pooled across all rooms for analysis and presented as attempted landings per house.

### Insecticide resistant phenotypes

The response of *Ae*. *aegypti* from Ticul to conventional type I and II pyrethroids (permethrin and deltamethrin) was assessed using standard Centers for Disease Control and Prevention (CDC) bottle bioassays [37]. Briefly, prior to the start of the intervention, mosquito eggs were collected from ovitraps installed in a random sample of 50 houses and used to initiate an F1 cohort of mosquitoes. The responses of three groups of 25 adult female mosquitoes (75 per insecticide) to diagnostic doses of permethrin (15 μg/bottle) and deltamethrin (10μg/bottle) were recorded as per CDC bottle assay protocols [38].

### Detection of kdr alleles

Genomic DNA extraction from field-caught mosquitoes was performed using Extracta DNA Prep for PCR–Tissue (QuantaBio, Beverly, MA). Individual whole mosquitoes were added to 25 μL of extraction reagent. Samples were incubated at 95°C for 20 min. Once cooled to room temperature, 25 μL of stabilization buffer was added to the samples, which were kept at -20°C until use. Allele-specific PCR methods were used to detect *kdr* mutations with known function. Genotypes were characterized using a CFX-96 RT-PCR system (BioRad, Hercules, CA) under specific cycling and melt curve conditions. Primers used were adopted from Saavedra-Rodriguez et al [39] for V1016I, Yanola et al [40] for F1534C and Saavedra-Rodriguez et al [41] for V410L. PCR reagents and conditions were based on Deming et al [42] and Saavedra-Rodriguez et al [39] for V1016I, Deming et al [42] for F1534C and Saavedra-Rodriguez et al [41] for V410L (see S3 Table).

### Community perceptions

Simple pre-deployment and post-deployment surveys were conducted across all households that received the metofluthrin emanators. The first survey was applied during the enrollment process, to detail the characteristics of the households (S1 Table) and record domestic mosquito control practices. During the second cycle of deployment, a follow-up survey posed exploratory, open questions about householder perceptions of the devices, the installation process, their impact and the acceptability of the intervention (S4 Table). The survey was applied to the 100 heads of households that consented to have the emanators deployed in their homes.

### Data analysis

The total number of *Ae*. *aegypti* adult males, females and blood-fed females were recorded across treatment and control arms for each entomological survey. In total, one pre-deployment baseline and seven post deployment treatment cycles were analyzed. To determine the level of statistical significance between treatment and control arms for each entomological indicator, generalized linear mixed models (GLMM) were run with a Poisson distribution and “house” as random effect. Abundance Rate Ratios (ARR), together with their 95% CI, were calculated for each entomological survey post-emanator installation. Based on the estimated ARR, efficacy was also calculated as the predicted percentage decrease of mosquitoes in the presence of the emanators [100 * (1-ARR)] [5]. Human landings by *Ae*. *aegypti* were calculated by averaging the number of adult *Ae*. *aegypti* landing over the 10-minute period in each room. All statistical analyses were performed using R programing (https://www.r-project.org/) and the *lme4* package [43].

## Results

The trial compared two treatments over 194 houses (n = 100 control, n = 94 treated) and seven post-treatment sampling periods (S1 Table). Six recruited treatment houses were lost due to the owners relocating. A total of 17,027 adult mosquitoes were collected during the trial. *Aedes aegypti* comprised 51.7% of the mosquitoes collected, with 52.4% of those being females, and 60.8% of females containing remnants of a blood-meal (Table 1).

**Table 1:** Total number of mosquitoes collected indoors during the trial.

	Control	Emanator	Totals
Total Mosquitoes	11,728	5,299	17,027
*Aedes aegypti*			
Total	6,048	2,760	8,808
Females	3,167	1,451	4,618
Blood-fed	1,898	909	2,807
Males	2,881	1,309	4,190
*Culex nigripalpus*			
Total	236	137	373
Females	148	89	237
Blood-fed	114	68	182
Males	88	48	136
*Culex quinquefasciatus*			
Total	5,444	2,402	7,846
Females	2,380	1,071	3,451
Blood-fed	1,521	685	2,206
Males	3,064	1,331	4,395

Collections took place between 30^th^ April and 31^st^ October 2018. Adult indoor resting mosquitoes were collected from all rooms within every house recruited to the trial. Two field collectors aspirated mosquitoes from each house for a total of 30 minutes using Prokopack aspirators. This was repeated for the baseline assessment and the 7 continuous deployment cycles.

**Fig 3:**
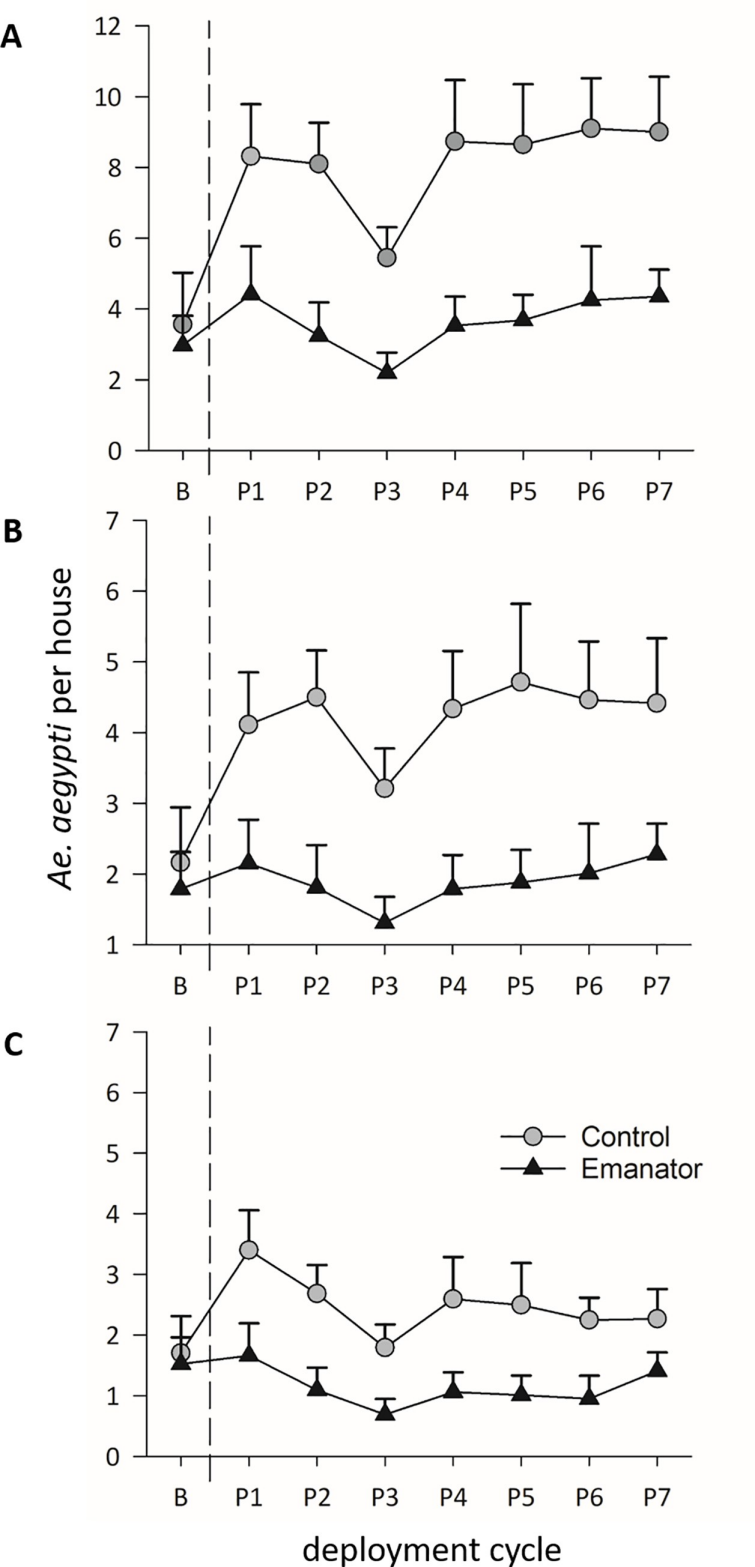
Influence of the metofluthrin emanators on indoor adult *Ae*. *aegypti* collections. Black triangles represent treated houses and grey circles represent controls for A) total *Ae*. *aegypti*, B) female *Ae*. *aegypti* and C) blood-fed *Ae*. *aegypti*. The dotted lines represent emanator deployment. The x-axis labels denote the pre-treatment baseline, and every subsequent deployment cycle (P1-P7). Data presented as means + 95% CI.

At baseline, no significant difference in *Ae*. *aegypti* adult indices was observed between arms (Table 2). Average (± SD) densities of adult *Ae*. *aegypti* per house in treatment and control arms were 2.97 (± 4.60) and 3.56 (± 7.64) respectively, for total adults 1.78 (± 2.90) and 2.16 (± 4.07) for females and 1.52 (± 2.42) and 1.70 (±3.18) for blood-fed females (Fig 3). After the metofluthrin emanators were deployed, adult indices remained at similar (pre-transmission season) values for treatment houses but increased markedly in the control arm (Fig 3). This difference between arms was statistically significant for all *Ae*. *aegypti* indices (Table 2 and Fig 3) for the duration of the trial.

**Table 2:** Abundance Rate Ratios (ARR) and their significance values (P) comparing entomological indices (A, B, C) between the treatment arms. The first row reports the results of the pre-deployment, baseline study. Each subsequent row indicates a sampling cycle post emanator replacement.

Survey	A: Total *Ae*. *aegypti*				B: Total Female *Ae*. *aegypti*				C: Total Blood-fed *Ae*. *aegypti*			
	ARR	CI (95%)	Z	P	ARR	CI (95%)	Z	P	ARR	CI (95%)	Z	P
Baseline	1	(0.598–1.703)	0.013	0.989	0.93	(0.541–1.607)	-0.275	0.783	1.04	(0.594–1.872)	0.147	0.883
P1	0.4	(0.267–0.581)	-4.704	<0.001	0.47	(0.323–0.672)	-4.082	<0.001	0.43	(0.281–0.638)	-4.086	<0.001
P2	0.35	(0.242–0.49)	-5.944	<0.001	0.38	(0.265–0.522)	-5.741	<0.001	0.43	(0.301–0.607)	-4.734	<0.001
P3	0.32	(0.222–0.454)	-6.276	<0.001	0.36	(0.254–0.513)	-5.691	<0.001	0.34	(0.218–0.504)	-5.148	<0.001
P4	0.35	(0.252–0.492)	-6.128	<0.001	0.36	(0.258–0.505)	-5.934	<0.001	0.38	(0.257–0.564)	-4.804	<0.001
P5	0.44	(0.315–0.612)	-4.882	<0.001	0.41	(0.285–0.592)	-4.785	<0.001	0.43	(0.276–0.667)	-3.763	<0.001
P6	0.36	(0.266–0.479)	-6.88	<0.001	0.38	(0.273–0.518)	-5.97	<0.001	0.35	(0.234–0.501)	-5.498	<0.001
P7	0.5	(0.376–0.647)	-4.935	<0.001	0.56	(0.408–0.754)	-3.793	0.001	0.64	(0.462–0.881)	-2.741	0.006

**Fig 4:**
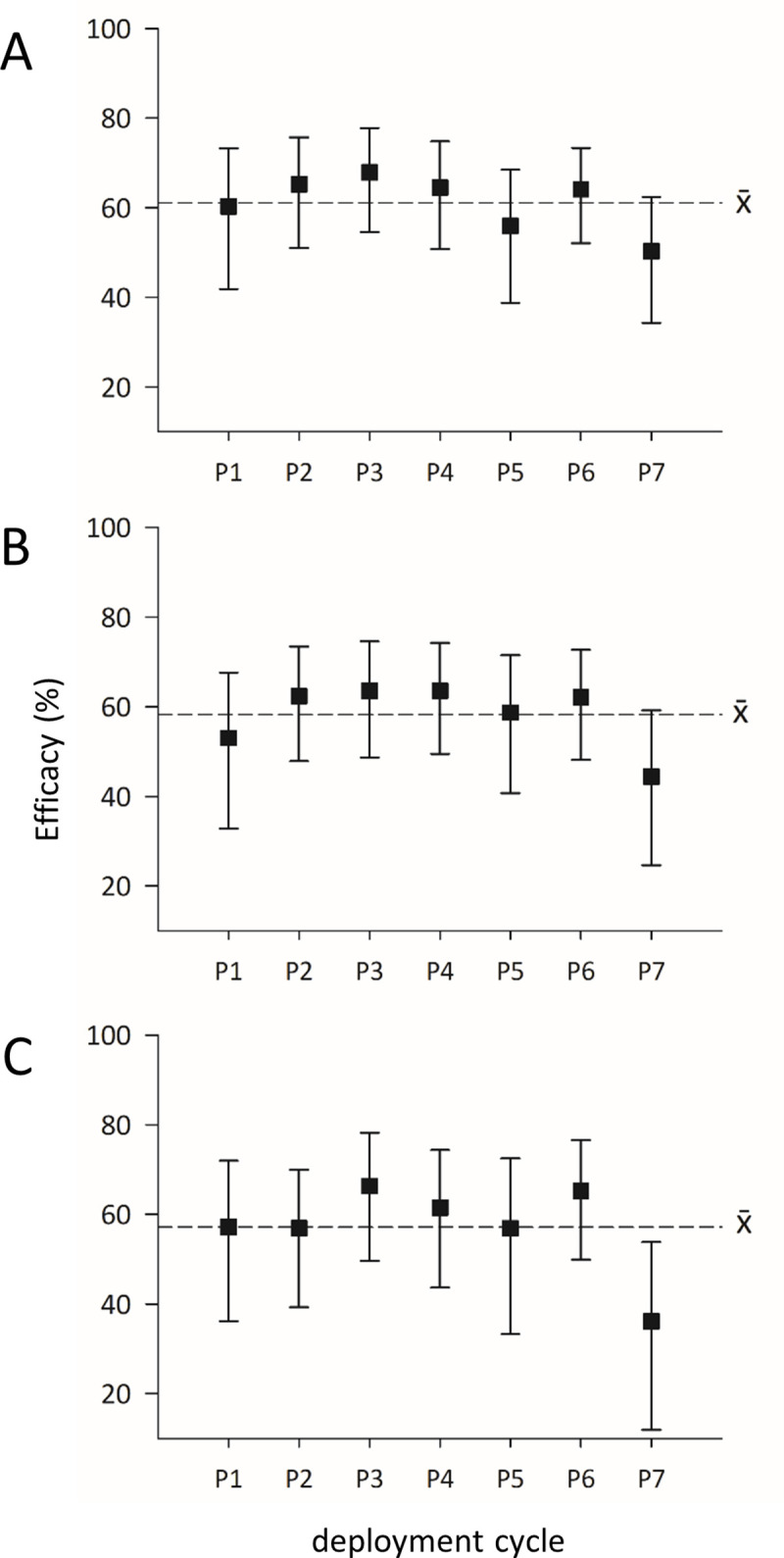
Efficacy of metofluthrin emanators against *Ae*. *aegypti* in comparison to baseline. Efficacy in relation to the untreated baseline for A) total *Ae*. *aegypti*, B) female *Ae*. *aegypti* and C) blood-fed *Ae*. *aegypti*. The dotted lines represent average efficacy for each measure. The x-axis labels denote successive deployment cycles (P1-P7). Data presented as means ± 95% CI.

Averaging ARR across all 7 deployment cycles demonstrates a reduction in *Ae*. *aegypti* abundance (estimated as 1/ARR) of 2.57 times for total numbers, 2.39 times for females and 2.33 times for female blood-fed mosquitoes (Table 2). The entomological impact of metofluthrin emanators as a percentage reduction of *Ae*. *aegypti* abundance is shown in Fig 4. The average reduction across all 7 post-exposure sampling dates was 60.19% (± 6.17) for total adults, 58.25% (± 7.14) for females, and 57.16% (± 10.10) for blood-fed females. The final sampling cycle (Post 7) had lower efficacy than the rest but the 95% confidence intervals overlapped across all sampling dates (Fig 4) indicating no statistical difference in effectiveness between cycles.

**Fig 5:**
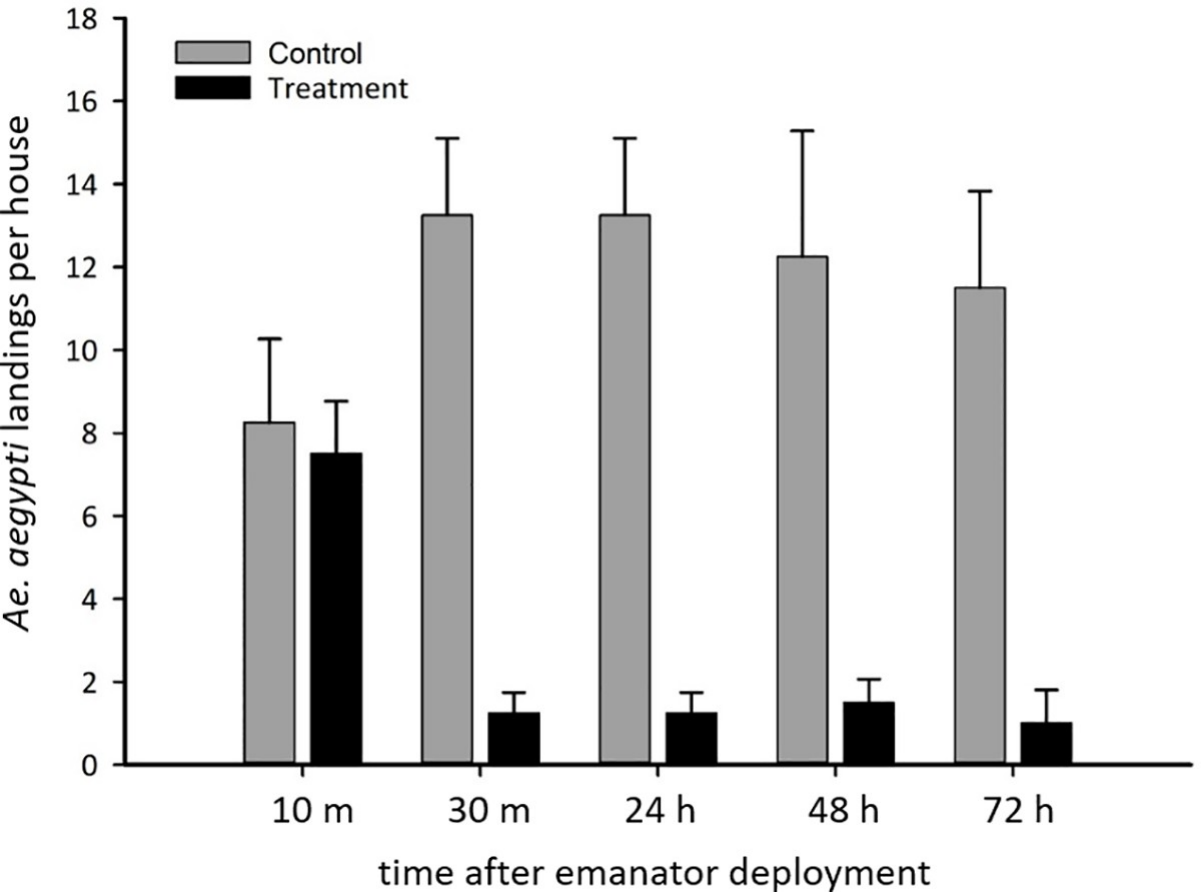
Impact of emanators on mosquito landing behaviour. *Ae aegypti* landings in the control (black bars) and treatment houses (grey bars). Pooled data from across both assessments is presented as means per house and 95% CI. These measures were made immediately following the first deployment of the emanators in a subset of recruited households (n = 16). Data for all rooms and houses were pooled and averaged for the control and treatment houses.

Metofluthrin emanators significantly reduced *Ae*. *aegypti* abundance, but they also had a large impact on *Ae*. *aegypti* landing attempts (Fig 5). Assessments of landing activity were conducted immediately after the first round of emanator installations. While the number of landings was unaffected at the initial 10 minute, post-deployment assessment (i.e. 95% CIs overlapped), observations over the subsequent 0.5–72 hrs, showed that metofluthrin emanators caused a highly significant, 90% reduction in landings (Fig 5).

**Fig 6:**
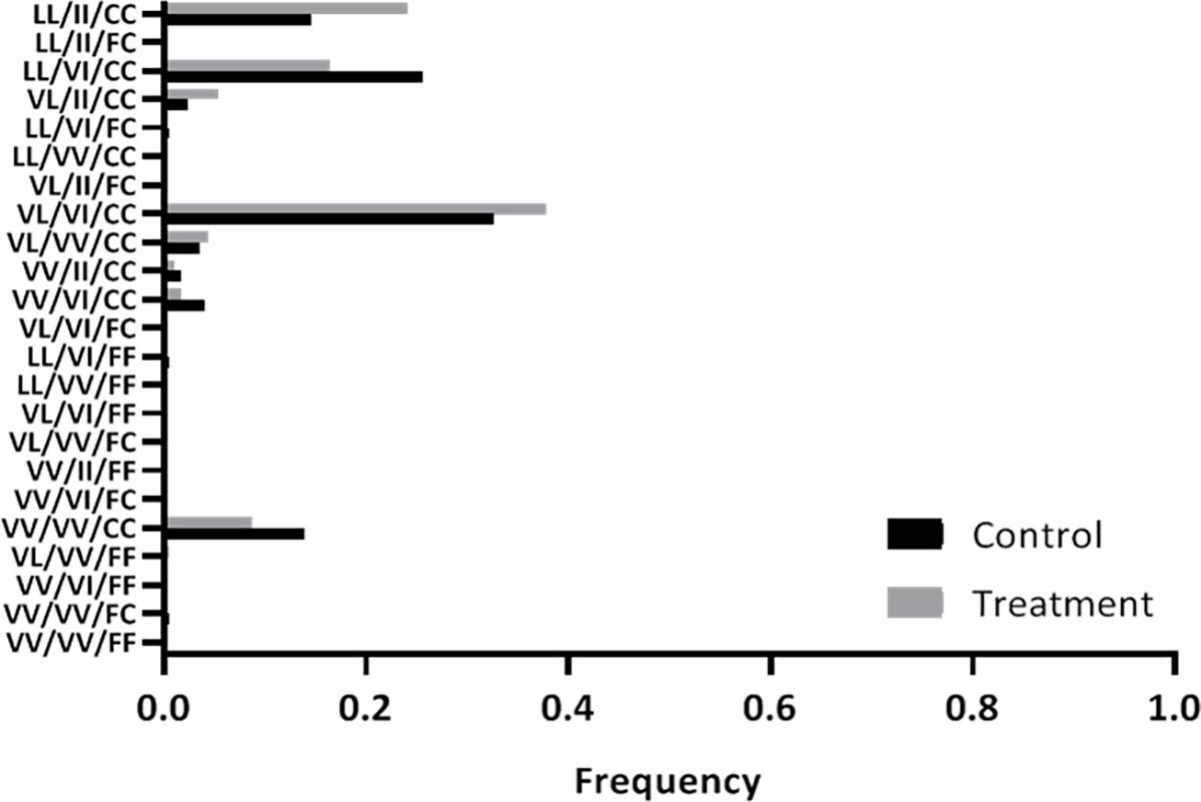
Frequencies of tri-locus genotypes of mosquitoes collected during the trial. The order of the genotypes on each row of the y axis is 410 / 1016 / 1534. The resistant mutations screened for were V410L, V1016I and F1534C. Resistant homozygous forms (RR) are denoted by LL, II and CC respectively. The triple susceptible genotype is at the bottom of the graph and the triple resistant genotype is at the top of the graph.

Current CDC bottle bioassay guidelines state that 97–100% mortality at the diagnostic time indicates susceptibility, 90%–96% mortality indicates that resistance is developing and <90% mortality implies resistance [38]. *Aedes aegypti* reared from our Ticul egg collections demonstrated a permethrin-resistant phenotype (87% knockdown) but a deltamethrin susceptible phenotype (98% knockdown). This was despite the high frequency of *kdr* mutations. A total of 3200 adult *Ae*. *aegypti*, collected from control and treatment houses, were tested for F1534C, V1016I, and V410L. F1534C was present in homozygous resistant (RR) form (ca. 95% of individuals tested carried this genotype) while homozygous (RR) forms of V1016I and V410L were also common (present in ca. 24% and 40% of individuals, respectively). Overall, there was no evidence of any change in allele frequency associated with treatment (Fig 6).

The pre-deployment enrolment surveys conducted across 200 households revealed two main drivers for accepting the metofluthrin emanators: to reduce mosquito numbers (50.5%) and to avoid mosquito-borne disease (35.5%). Before receiving the intervention, most people reported the use of consumer products against mosquitoes: 36% used repellents, 61% used insecticides, 40% used plug-in devices, and 20% used coils. During the enrolment process, after the installation process was demonstrated, we asked householders which indoor spaces would be most suitable for the deployment of emanators. Bedrooms (43.5%), living rooms (15.5%), kitchens (13.5%), bathrooms (12%) and dining areas (3%) were identified.

After the second deployment cycle, all 100 heads of households from the treated houses (none had left the study at this time) were asked open-ended questions about their perceptions of the emanators (S4 Table). The majority (85%) felt that the product protected against mosquito bites. When asked to list characteristics of the emanators most responders listed positive attributes of the devices (83%). The most commonly stated descriptors included observations that the emanators were “odorless”, “unobtrusive”, “environmentally friendly”, “easy to install” and “safe for the family and pets”. The one negative issue identified by participants was related to the process of hanging the emanators from ceilings which was perceived to be time-consuming and impractical (12%). Almost all household heads (95%) thought that the intervention was suitable for wider use in the community and 85% said that they might be willing to pay for the emanators (dependent on price).

## Discussion

This is the first large-scale randomized control trial to evaluate the entomological impact of metofluthrin emanators on urban *Ae*. *aegypti*. Other entomological and epidemiological trials targeting the same species with a related molecule, transfluthrin, are underway in Iquitos, Peru [15]. Our trial builds on a variety of observations in laboratory and semi-field settings [13,16,19,33,44, 45] to confirm that the deployment of metofluthrin emanators indoors can have a sustained and significant impact on *Ae*. *aegypti* population densities and their biting behaviours in urban environments. These effects were evident despite the presence of multiple pyrethroid-resistance alleles in the local *Ae*. *aegypti* population. Our assessments were made in the absence of any other vector control activity. During the trial period, the Sistema Nacional de Vigilancia Epidemiológica (SINAVE) reported just six laboratory-confirmed cases of Zika and one dengue case from Ticul. These did not trigger a vector control response by the MOH and none of these cases were in houses recruited to our trial.

There are many consumer products that release volatile insecticides to repel or kill mosquitoes. These include candles, coils, plug-ins, passive emanators, battery-operated fans and impregnated nets or fabrics [36]. In relation to public health, the “spatial repellent” paradigm that exploits volatile chemicals to reduce mosquito numbers indoors is not new [46] but it has recently been re-visited because of a pressing need for effective complements and alternatives to indoor residual sprays, space sprays and treated bed nets [16]. In that context, the concept of using chemicals in vapour phase to create bite free protected areas is a popular one [13,16,17,19,47]. Two of the most discussed molecules at the present time are the polyfluorinated synthetic pyrethroids transfluthrin and metofluthrin. Both have high vapour pressures and are suitable for formulation in devices that facilitate volatilization at room temperature. Although the recent literature focuses on their “spatial repellent” impacts rather than direct toxicity, the balance of behavioural and lethal effects is a function of dose. At higher doses, in vapour phase, metofluthrin and transfluthrin cause knockdown and death [13,48].

Devices that release chemical passively, at room temperature, are a focus of current public health interests because of their portability, potential low cost, and suitability for deployment in resource-poor environments. Examples include transfluthrin-treated plastics [12], metofluthrin treated paper concertinas [36,45] and metofluthrin-impregnated polyethylene mesh [13]. It is a derivation of the latter 10% w/w metofluthrin mesh that was deployed during this study. Our simple post-deployment survey, despite being conducted in only the second deployment cycle, suggested that the community viewed the emanators favourably. A major limitation of this survey was that the study was not blinded and so we cannot discount response bias among the participants.

Metofluthrin emanators deployed at a rate of one device per room and replaced at three-week cycles reduced *Ae*. *aegypti* abundance indoors by 60% in comparison to untreated houses. This effect was consistent throughout the entire transmission season (June to October). While it is difficult to determine if the reduction in vector abundance was due to a behavioural impact (i.e. repellent, deterrent or confusant effects), its killing power, or both, the effect of deployment was highly significant. We did not measure mortality, or dispersal. Nonetheless, the observed reduction in vector abundance is comparable to that reported for other insecticidal methods with proven epidemiological impacts such as indoor space spraying [3] and targeted indoor residual spraying (TIRS) [49]. Our data demonstrate that metofluthrin deployment also affects mosquito landing behaviour, with a 90% reduction in activity.

Traditional models of vectorial capacity demonstrate that the greatest epidemiological impacts of vector control tools occur when both adult survival and biting rates are affected [50]. However, there are considerable subtleties involved in predicting the effect of volatile pyrethroids that disrupt mosquito behaviour and that may have complex effects on survival [51,52]. For example, interventions that permit significant mosquito survival or that prevent mosquitoes taking full blood meals may increase the proportion of potentially infectious mosquitoes in the population or encourage multiple, partial blood feeds [20,53,54]. Moreover, while some perturbations to behaviour may be desirable, others are problematic. A major concern about repellent interventions is that, unless coverage is universal, the burden of transmission will shift to unprotected neighbours [20,53,55]. Interestingly, true repellency does not appear to be a major consequence of exposure to the 10% w/w metofluthrin device [13,36] but this may simply be a result of the relatively high dose of metofluthrin in the formulation. Incorporating the complexity of volatile insecticide effects into epidemiological models will be a key step in predicting how different emphases on bite disruption, repellency and mortality will combine to impact ABV transmission at the community level.

Another key issue that determines the utility of metofluthrin and transfluthrin as tools for *Ae*. *aegypti* control is their impact on mosquito populations with high frequencies of alleles that confer resistance to conventional pyrethroids. This is a global challenge [56] that affects many insecticide-based vector control operations [5]. Resistance to pyrethroids is mediated by a range of mechanisms that involve point mutations in specific genes or upregulation of metabolic enzymatic pathways [8]. For metofluthrin and the related molecule, transfluthrin, there is a poor understanding of the impact of conventional pyrethroid resistance mechanisms on efficacy [24,25] and the volatility of the active ingredient has challenged the development of simple phenotypic bioassays for susceptibility. In our study, we used CDC bottle bioassays and screens for *kdr* alleles to describe pyrethroid resistance in the trial population. The mutation F1534C was ubiquitous as a homozygous resistant genotype in Ticul while the homozygous and heterozygous resistant forms of V1016I and V410L were also common. All three homozygous resistant alleles commonly co-exist and of these, F1534C and V410L are strongly associated with phenotypes that resist type I or type II pyrethroids [57–60]. Despite this, the CDC bottle assays performed at the outset of our trial demonstrated a permethrin-resistant but deltamethrin-susceptible phenotype. The reason for the disjunct between phenotype and genotype is not clear, although kdr mutations are only partly responsible for pyrethroid resistance [59]. It is evident however, that the metofluthrin emanator, when deployed at a rate of one device per room, and replaced every three weeks, is highly effective against *Ae*. *aegypti* carrying high frequencies of kdr alleles.

Allele frequencies did not differ significantly between the control and treatment arms and, as most mosquitoes collected by our sequential removal sampling protocols are alive [35], this strongly suggests that the kdr mutations present in mosquitoes surviving metofluthrin treatment were not subject to any obvious selection pressure. It should be noted, however, that our trial did not deploy the emanators at high coverage and that selection pressures will increase with more widespread, contiguous deployment. Although spatial repellents are sometimes presented as less vulnerable to the evolution of resistance than more conventional insecticides [16], behavioural disruption will also affect survival by disturbing optimal resting and feeding behaviours. Even sublethal effects on survival exert significant selection pressure and facilitate adaptive mutations [61].

This trial delivers an important proof of principle regarding the vector control potential of volatile insecticides that offer fast coverage of indoor spaces to protect residents against the bites of *Ae*. *aegypti*. As an outbreak response tool, the distribution of metofluthrin emanators may address the low coverage that challenges other vector control approaches, providing a less intrusive, more easily deployable tool for integrated vector management. We recognize that the metofluthrin emanators will mitigate arbovirus outbreaks only if they can be deployed for epidemiologically significant periods at a high coverage. That will be difficult without some element of community development and ownership, particularly in complex urban landscapes. Ultimately, a robust field assessment of community acceptance and community-led implementation of the emanators may provide an opportunity to integrate these rapidly deployable and safe insecticidal formulations as elements of community-based programs [47,62].

## Supporting information

S1 Table Baseline construction characteristics of houses. At the recruitment phase, after assigning households to the control or treatment arms, details of house construction were collected. Characteristics were largely similar across all households.
(DOCX)

S2 Table Range of dates over which the entomological collections occurred. Baseline refers to the pre-deployment survey, Post 1–7 refer to the subsequent surveys conducted during successive deployments of the emanators. Metofluthrin emanators had a 3-week replacement cycle and were deployed 2–5 days prior to the beginning of each survey.
(DOCX)

S3 Table Modifications of previously published PCR conditions used to screen for kdr mutations. PCR conditions were based on Deming et al [40] and Saavedra-Rodriguez et al [37] for V1016I, Deming et al [40] for F1534C and Saavedra-Rodriguez et al [39] for V410L.
(DOCX)

S4 Table Open-ended survey questions for heads of households. Some basic information was collected during enrollment of households and after deployment of the emanators. Pre-treatment questions were asked of all households (n = 200). The questions asked during the second deployment cycle were asked of all head of households from treated houses (n = 100).
(DOCX)
