# Supplementary material for: The entomological impact of passive metofluthrin emanators against indoor Aedes aegypti: A randomized field trial
Source: PLoS Negl Trop Dis. 2021 Jan 26;15(1):e0009036. doi: 10.1371/journal.pntd.0009036 (PMC7864418; doi:10.1371/journal.pntd.0009036)
Supplement: S1 Table — At the recruitment phase, after assigning households to the control or treatment arms, details of house construction were collected. Characteristics were largely similar across all households. (DOCX) [file pntd.0009036.s001.docx]

**Supplementary material**

**Table S1:** Baseline construction characteristics of houses

| Variable | Category | Treatment houses (n=50) | | Control houses (n=50) | |
| --- | --- | --- | --- | --- | --- |
|  |  | n (± se) | range or % | n (± se) | range or % |
| General | *x̄ people per house* | 5.9 (±3.5) | 2-19 | 5.5 (±4.3) | 2-28 |
|  | *x̄ rooms per house* | 4.6 (±2.1) | 2-10 | 4.1(±2.5) | 1-16 |
|  | *Single floor level* | 48 | 96.0 | 50 | 100.0 |
|  | *Front or back yard* | 36 | 72.0 | 39 | 78.0 |
| Floor | *Mixed* | 19 | 38.0 | 20 | 40.0 |
|  | *Concrete* | 17 | 34.0 | 19 | 38.0 |
|  | *Tile* | 14 | 28.0 | 11 | 22.0 |
| Roof | *Mixed* | 26 | 52.0 | 24 | 48.0 |
|  | *Concrete* | 21 | 42.0 | 20 | 40.0 |
|  | *Zinc* | 1 | 2.0 | 1 | 2.0 |
|  | *Metal* | 1 | 2.0 | 3 | 6.0 |
|  | *Palm thatch (huano)* | 1 | 2.0 | 2 | 4.0 |
| Walls | *Concrete* | 40 | 80.0 | 37 | 74.0 |
|  | *Mixed* | 8 | 16.0 | 10 | 20.0 |
|  | *Wood* | 1 | 2.0 | 2 | 4.0 |
|  | *Mud wall (bajereque)* | 1 | 2.0 | 1 | 2.0 |
| Water supply | *Piped water* | 49 | 98.0 | 47 | 94.0 |
|  | *Well water* | 1 | 2.0 | 3 | 6.0 |
| Water storage | *Yes* | 24 | 48.0 | 28 | 56.0 |
|  | *No* | 26 | 52.0 | 22 | 44.0 |
| Reason for water storage | *Washing* | 19 | 79.2 | 21 | 75.0 |
|  | *Animal consumption* | 3 | 12.5 | 3 | 10.7 |
|  | *Cleaning the house* | 1 | 4.2 | 2 | 7.1 |
|  | *Drinking water* | 1 | 4.2 | 1 | 3.6 |
|  | *Other* | 0 | - | 1 | 3.6 |
| Electricity | *Yes* | 50 | 100 | 50 | 100 |
|  | *No* | 0 | - | 0 | - |
