# Supplementary material for: The entomological impact of passive metofluthrin emanators against indoor Aedes aegypti: A randomized field trial
Source: PLoS Negl Trop Dis. 2021 Jan 26;15(1):e0009036. doi: 10.1371/journal.pntd.0009036 (PMC7864418; doi:10.1371/journal.pntd.0009036)
Supplement: S2 Table — Baseline refers to the pre-deployment survey, Post 1–7 refer to the subsequent surveys conducted during successive deployments of the emanators. Metofluthrin emanators had a 3-week replacement cycle and were deployed 2–5 days prior to the beginning of each survey. (DOCX) [file pntd.0009036.s002.docx]

**Supplementary material**

**Table S2.** Range of dates over which the entomological collections occurred.

| **Control** | **Start** | **End** |
| --- | --- | --- |
| Baseline | 30-Apr-2018 | 22-May-2018 |
| Post 1 | 28-May-2018 | 28-Jun-2018 |
| Post 2 | 9-Jul-2018 | 13-Jul-2018 |
| Post 3 | 30-Jul-2018 | 8-Aug-2018 |
| Post 4 | 22-Aug-2018 | 29-Aug-2018 |
| Post 5 | 11-Sep-2018 | 19-Sep-2018 |
| Post 6 | 2-Oct-2018 | 12-Oct-2018 |
| Post 7 | 23-Oct-2018 | 31-Oct-2018 |
