# Supplementary material for: The entomological impact of passive metofluthrin emanators against indoor Aedes aegypti: A randomized field trial
Source: PLoS Negl Trop Dis. 2021 Jan 26;15(1):e0009036. doi: 10.1371/journal.pntd.0009036 (PMC7864418; doi:10.1371/journal.pntd.0009036)
Supplement: S3 Table — PCR conditions were based on Deming et al [40] and Saavedra-Rodriguez et al [37] for V1016I, Deming et al [40] for F1534C and Saavedra-Rodriguez et al [39] for V410L. (DOCX) [file pntd.0009036.s003.docx]

**Supplementary material**

**Table S3.** Modifications of previously published PCR conditions used to screen for kdr mutations.

| **V1016I** | **F1534C** | **V410L** |
| --- | --- | --- |
| 0.5 µM of each forward and reverse primer (Val1016_fwd and Ile1016_fwd; Ile1016_rev, respectively) were added to a total reaction volume of 25µL containing 10µL SYBR Green Supermix (BioRad), 6µL ddH2O and 1µL genomic DNA. The PCR thermocycling conditions consisted of 3min at 95°C, 40 cycles of 10 sec at 95°C, 10 sec at 60°C, 30 sec at 72°C followed by 10 sec at 95°C and a melting curve from 65°C to 95 °C with increments of 0.2°C every 10 sec. | 0.325 µM of each forward and reverse primer (Phe1534_fwd and Cys1534_fwd; 1534_rev, respectively) were added to a total reaction volume of 25µL containing 9µL SYBR Green Supermix (BioRad), 7.15µL ddH2O and 2µL genomic DNA. The PCR thermocycling conditions consisted of 3 min at 95°C, 37 cycles of 10 sec at 95°C, 10 sec at 57°C, 30 sec at 72°C followed by 10 sec at 95°C and a melting curve from 65°C to 95 °C with increments of 0.5°C every 5 sec. | As previously published [39] with no protocol modifications |
