# Supplementary material for: The entomological impact of passive metofluthrin emanators against indoor Aedes aegypti: A randomized field trial
Source: PLoS Negl Trop Dis. 2021 Jan 26;15(1):e0009036. doi: 10.1371/journal.pntd.0009036 (PMC7864418; doi:10.1371/journal.pntd.0009036)
Supplement: S4 Table — Some basic information was collected during enrollment of households and after deployment of the emanators. Pre-treatment questions were asked of all households (n = 200). The questions asked during the second deployment cycle were asked of all head of households from treated houses (n = 100). (DOCX) [file pntd.0009036.s004.docx]

**Supplementary material**

**Table S4:** Open-ended survey questions for heads of households

| **Pre-deployment / enrollment** |
| --- |
| What is your main reason for participating in this study? |
| What do members of your household use to control mosquitoes? |
| In which rooms in this house would you put the emanators? |
| **Post-deployment (second cycle)** |
| Did the process of installation have any positive or negative characteristics? |
| After the emanators were installed, did you perceive any difference in the amount of mosquito bites? |
| Did the emanator have any positive or negative characteristics? |
| Do you think that emanators should be installed in more houses in the community? |
| If the emanators were available in local shops, would you pay for the product? |
